# MED10 Drives the Oncogenicity and Refractory Phenotype of Bladder Urothelial Carcinoma Through the Upregulation of hsa-miR-590

**DOI:** 10.3389/fonc.2021.744937

**Published:** 2022-01-13

**Authors:** Chia-Chang Wu, Yuan-Hung Wang, Su-Wei Hu, Wen-Ling Wu, Chi-Tai Yeh, Oluwaseun Adebayo Bamodu

**Affiliations:** ^1^ Department of Urology, Shuang Ho Hospital, Taipei Medical University, New Taipei City, Taiwan; ^2^ Taipei Medical University (TMU) Research Center of Urology and Kidney, Taipei Medical University, Taipei City, Taiwan; ^3^ Department of Urology, School of Medicine, College of Medicine, Taipei Medical University, Taipei City, Taiwan; ^4^ Graduate Institute of Clinical Medicine, College of Medicine, Taipei Medical University, Taipei, Taiwan; ^5^ Department of Medical Research, Shuang Ho Hospital, Taipei Medical University, New Taipei City, Taiwan; ^6^ Department of Medical Laboratory Science and Biotechnology, Yuanpei University of Medical Technology, Hsinchu City, Taiwan; ^7^ Department of Hematology and Oncology, Cancer Center, Shuang Ho Hospital, Taipei Medical University, New Taipei City, Taiwan

**Keywords:** bladder urothelial carcinoma, MED10, hsa-miR-590, metastasis, cancer stemness, disease progression, recurrence, therapy failure

## Abstract

**Background:**

Dysfunctional transcription machinery with associated dysregulated transcription characterizes many malignancies. Components of the mediator complex, a principal modulator of transcription, are increasingly implicated in cancer. The mediator complex subunit 10 (MED10), a vital kinase module of the mediator, plays a critical role in bladder physiology and pathology. However, its role in the oncogenicity, metastasis, and disease recurrence in bladder cancer (BLCA) remains unclear.

**Objective:**

Thus, we investigated the role of dysregulated or aberrantly expressed MED10 in the enhanced onco-aggression, disease progression, and recurrence of bladder urothelial carcinoma (UC), as well as the underlying molecular mechanism.

**Methods:**

Using an array of multi-omics big data analyses of clinicopathological data, *in vitro* expression profiling and functional assays, and immunocytochemical staining, we assessed the probable roles of MED10 in the progression and prognosis of BLCA/UC.

**Results:**

Our bioinformatics-aided gene expression profiling showed that MED10 is aberrantly expressed in patients with BLCA, is associated with high-grade disease, is positively correlated with tumor stage, and confers significant survival disadvantage. Reanalyzing the TCGA BLCA cohort (n = 454), we showed that aberrantly expressed MED10 expression is associated with metastatic and recurrent disease, disease progression, immune suppression, and therapy failure. Interestingly, we demonstrated that MED10 interacts with and is co-expressed with the microRNA, hsa-miR-590, and that CRISPR-mediated knockout of MED10 elicits the downregulation of miR-590 preferentially in metastatic UC cells, compared to their primary tumor peers. More so, silencing MED10 in SW1738 and JMSU1 UC cell lines significantly attenuates their cell proliferation, migration, invasion, clonogenicity, and tumorsphere formation (primary and secondary), with the associated downregulation of BCL-xL, MKI67, VIM, SNAI1, OCT4, and LIN28A but upregulated BAX protein expression. In addition, we showed that high MED10 expression is a non-inferior biomarker of urothelial recurrence compared with markers of cancer stemness; however, MED10 is a better biomarker of local recurrence than any of the stemness markers.

**Conclusion:**

These data provide preclinical evidence that dysregulated MED10/MIR590 signaling drives onco-aggression, disease progression, and recurrence of bladder UC and that this oncogenic signal is therapeutically actionable for repressing the metastatic/recurrent phenotypes, enhancing therapy response, and shutting down stemness-driven disease progression and relapse in patients with BLCA/UC.

## Introduction

Bladder cancer (BLCA), with 573,278 new cases in 2020 and a projected 72.9% increase in incidence by 2040, ranks as one of the most diagnosed malignancies and a leading cause of cancer-associated mortality for both sexes and all ages, globally (1). Cigarette smoking, male sex, and advanced age contribute to the development of BLCA (1, 2). BLCA is characterized by high inter- and intra-tumor heterogeneity; more than 90% of BLCA cases are in some form transitional cell or urothelial, histologically, and localized bladder urothelial carcinoma (UC), occurring anywhere from the renal pelvis to the urethra, may either be non-muscle invasive (NMIBC, T1 stage) or muscle-invasive bladder cancer (MIBC, T2–T4), with ~3 in every 4 BLCA presenting as NMIBC (3–5). More so, up to 70% of all NMIBCs recurs and an estimated 20% progresses to MIBC, regardless of local therapy, with a persisting dismal prognosis for MIBC, which continues to be characterized by less than a 50% 5-year survival rate (4). Currently, treatment modality for BLCA includes cystoscopic surveillance, intravesical therapy, chemotherapy and/or radiation therapy, and immunotherapy; however, radical cystectomy remains the treatment of choice despite the risk of enhanced morbidity or probable overtreatment of low-risk patients (2, 6, 7). Diagnosis-wise, over the last decade, liquid biopsy including various urine-associated biomarkers, such as DNA methylation and mutations, protein-based assays, mRNA, and non-coding RNA signatures, has been touted as a clinically effective modality for patient selection, and a bio-tool for precision medicine, informing therapy choice and real-time monitoring of therapeutic effects (8). Moreover, there is accruing evidence of the exploitable role of circulating tumor cells (CTCs) as biomarkers of diagnosis and prognosticators of disease recurrence, progression, and poor survival in patients with BLCA/UC (9). Howbeit, the high incidence of recurrent NMIBC and poor survival rate of MIBC, despite these advances in diagnostic (8, 9) and therapeutic strategies (6, 7), necessitate the discovery and characterization of novel actionable molecular targets and development of new therapeutic approaches.

Transcription constitutes a vital part of the bio-cellular processes essential for protein production in eukaryotic cells, as such transcription is usually (not always) enhanced in cancerous cells, to facilitate their increased metabolic activity and proliferation (10). This highlights the exploitability of the transcriptional activity in malignant cells as a probable anticancer therapeutic strategy. The mediator of RNA polymerase II transcription subunit 10 (MED10) is a middle component of the highly conserved tetramodular (head, middle, tail, and cyclin-dependent kinase) Mediator complex in humans, and reports indicate that the Mediator complex serves as “a bridge between regulatory proteins and RNA polymerase II (Pol II), thereby regulating the Pol II-dependent transcription” (11). There is accruing evidence that altered expressions of components of the Mediator complex play important roles in tumor initiation and disease progression; however, these reports are largely divergent. Zhao et al. reported a high expression of MED15 in breast cancer tissues with enhanced TGFβ/Smad3 signaling, while inhibiting MED15 suppressed the metastatic potential of a highly aggressive breast cancer cell line (12). Conversely, the decreased expression of MED15 protein is implicated in uterine leiomyosarcomas regardless of mutational status (13) and MED15 is considered a tumor suppressor in oral/oropharyngeal cancers (14). In contrast, while MED1 expression is downregulated and inversely correlated with the expression of metastasis-related genes in melanoma (15), lung cancer (16, 17), and bladder cancer (18), enhanced MED1 activity has been reported in prostate and breast cancer, likely due to its function as a hub for nuclear hormone receptors (18, 19). Moreover, in spite of reports indicating that cyclin-dependent kinase (CDK)8-Mediator module is an oncogene, several studies support the tumor-suppressor role of CDK8, under certain conditions (20). This functional diversity of the Mediator and their divergent roles in different cancer types pique research interest and, howbeit controversial, may be exploited for the discovery of a surrogate biomarker of disease progression or development of inhibitors targeting candidate Mediator. Against the background of these contradictory reports, and the yet unknown role of MED10 in BLCA, the present study investigated the probable role of dysregulated or aberrantly expressed MED10 in the enhanced onco-aggression, disease progression, and recurrence of bladder UC, as well as the underlying molecular mechanism.

## Material and Methods

### Cell Culture and Chemicals

The normal human primary bladder epithelial BdEC (ATCC^®^ PCS-420-010™) cell line was obtained from the ATCC (American Type Culture Collection, Manassas, VA, USA), and the human bladder transition cell carcinoma cell lines SW1738 and JMSU1 were kind gifts from CTY (Taipei Medical University - Shuang Ho Hospital, New Taipei City, Taiwan) and cultured in RPMI 1640 (Thermo Fisher Scientific Inc., Bartlesville, OK, USA). Culture medium was supplemented with 10% fetal bovine serum (FBS, #26140079, Thermo Fisher Scientific Inc., Bartlesville, OK, USA) and 100 U/ml of penicillin–streptomycin (Thermo Fisher Scientific Inc., Bartlesville, OK, USA). All cells used in the study were not greater than passage number 3 (≤P.3). Cells were subcultured at ≥98% confluence or culture media changed every 48 h. Stock solutions of 100 mM in 0.01% DMSO were stored at -20°C, until use.

### Antibodies

Monoclonal antibodies against MED10 (C-2: #sc-393450), BAX (B-9: #sc-7480), BCL-xL (H-5: #sc-8392), Ki67 (#sc-23900), Vimentin (V9: #sc-6260), and SNAI1 (G-7: #sc-271977) were obtained from Santa Cruz Biotechnology (Dallas, TX, USA); OCT4A (#2840) and LIN28A (#8706) were purchased from Cell Signaling Technology, Inc. (CST, Beverly, MA, USA); and GAPDH (#sc-32233) was from Santa Cruz Biotechnology (Santa Cruz, CA, USA).

### Colorimetric Cell Proliferation Assay

For cell proliferation, Invitrogen alamarBlue™ high-sensitivity cell viability reagent (#A50100, Thermo Fisher Scientific Inc., Bartlesville, OK, USA) was used strictly following the manufacturer’s instruction. Briefly, after 1 × 10^3^ wild-type (WT) or MED10-silenced (shMED10) SW1738 or JMSU1 cell lines were seeded per well in triplicates with three biological replica for each assay in 96-well microtiter plates containing supplemented growth media and incubated at 37°C in humidified 5% CO_2_. After 24 h, the cells were incubated with alamarBlue™ for 2 h at 37°C. The number of dye-stained viable proliferating cells was read at 570-nm absorbance wavelength in the Molecular Devices SpectraMax M3 multimode microplate reader (Molecular Devices LLC., San Jose, CA, USA).

### Silencing MED10 by RNA Interference

MED10-Human, 4 unique 29mer shRNA constructs in lentiviral GFP vector containing pGFP-C-shLenti (#TL303299; MED10 Human shRNA Plasmid Kit (Locus ID 84246; OriGene Technologies Inc., Rockville, MD, USA) were packaged and transfected into SW1738 or JMSU1 cells to silence MED10. Non-effective 29-mer scrambled shRNA cassette in pGFP-C-shLenti Vector, TR30021, served as negative control. Stably transfected monoclonal SW1738 or JMSU1 cells were selected using 2 μg/ml puromycin, as recommended by the manufacturer. The effect of the lentiviral infection was enhanced by adding Sigma-Aldrich^®^ polybrene (#TR-1003, Merck KGaA, Darmstadt, Germany). MED10 knockdown in the cells was verified by Western blotting and quantitative real-time PCR. The shRNA sequences for MED10 are as follows: shMED10#1 5′-GACAGCAGCTTCATGATATTA-3′, and shMED101#2 5′-ATCGACACCATGAAGAAATTT-3′.

### MED10 Ectopic Expression

We overexpressed MED10 in BdEC cells by transfecting the human MED10 (NM_032286.2) cDNA sequence cloned into pCMV6-Entry vector (pCMV-MED10; #V0529, GeneCopoeia, Inc., Rockville, MD, USA) using Lipofectamine™ LTX with PLUS™ reagent (#15338100, Life Technologies, Thermo Fisher Scientific Inc., NY, USA). BdEC cells were seeded and cultured in 35-mm-diameter dishes till they attained 60% confluence. On transfection day, 1 mg of DNA diluted in 100 μl of serum-free medium and 6 μl of Lipofectamine™ LTX with PLUS™ reagents were then added. The DNA-PLUS mix was incubated at room temperature for 20 min, and 4 μl of Lipofectamine reagent was added, followed by an additional 20-min incubation. After incubation, the BdEC cells were carefully washed twice with serum-free media and 800 μl of serum-free transfection medium. The cells were then incubated with DNA-PLUS–Lipofectamine reagent mix in 5% humidified CO_2_ incubator at 37°C for 3 h. Thereafter, recovery medium with 10% FBS was added to a final volume of 2 ml and incubated overnight. After this, the recovery medium was suctioned and fresh RPMI 1640 medium containing serum and antibiotics was added.

### Bladder Cancer Tissue Samples

A total of 79 archived tissue specimens diagnosed with bladder urothelial carcinoma (UC) were retrieved from the Department of Pathology at Taipei Medical University–Shuang Ho Hospital. These samples were originally collected for routine clinical diagnostic purposes and subsequently preserved in the hospital’s pathology archives. As the research was retrospective and involved the analysis of previously collected, fully de−identified specimens, the Institutional Review Board (IRB) of Taipei Medical University reviewed the protocol. It addressed a related non−compliance (NC) event. Upon review, the IRB concluded that the non−compliance of this study was appropriately managed and that corrective measures were implemented in accordance with ethical and regulatory standards governing human subject research.

### Immunohistochemical and Immunofluorescence Staining Assays

Immunohistochemical (IHC) staining was performed on formalin-fixed paraffin-embedded (FFPE) samples from our BLCA/UC cohort (n = 79) consisting benign (n = 21), T1 (n = 11), T2 (n = 17), T3 (n = 9), T4 (n = 5), and metastatic (M1, n = 16) bladder UC cases. Samples were probed with primary antibodies against MED10, MKI67/Ki67, OCT4, and LIN28A at 1:200 dilution following standard IHC protocol. Protein expression was scored by two independent pathologists using the quick-score (Q-score) formula Q = I × P, where I is staining intensity [0 (no staining), 1+ (weak), 2+ (moderate), and 3+ (strong)] and P represents percentage of stained cells. Maximum Q-score = 300. For immunofluorescence (IFC) staining, WT or shMED10 tumorspheres derived from corresponding SW1738 and JMSU1 cells were fixed with 4% paraformaldehyde after they were plated onto poly-*L*-lysine-coated glass coverslips, then they were washed carefully with cold PBS thrice, permeabilized with 0.1% Triton X-100/PBS solution for 10 min, and then incubated with primary antibodies against OCT4 and LIN28A at 1:400 dilution, followed by Cy5-labeled goat anti-mouse Alexa Fluor 488 secondary antibodies (#R37120, Thermo Fisher Scientific Inc.) for 1 h. DAPI (4′,6-diamidino-2-phenylindole; #D1306, Molecular Probes, Thermo Fisher Scientific Inc.) was used for nuclear staining. Cell visualization and imaging were performed using the Nikon E800 fluorescent microscope (Nikon Instruments Inc., Melville, NY, USA).

### Western Blotting Assay

Protein blots derived from 20 µg of WT or shMED10 UC cell protein samples and separated by 10% sodium dodecyl sulfate polyacrylamide gel electrophoresis (SDS-PAGE) were transferred onto polyvinylidene fluoride (PVDF) membranes using the Bio-Rad Mini-Protein electro-transfer system (Bio-Rad Laboratories, Inc., Hercules, CA, USA). The PVDF membranes were blocked for 1 h with 5% non-fat milk in Tris-buffered saline with Tween 20 (TBST) and then probed overnight at 4°C with primary monoclonal antibodies against MED10 (1:1,000, Santa Cruz), BAX (1:1,000, Santa Cruz), BCL-xL (1:1,000, Santa Cruz), MKI67/Ki67 (1:1,000, Santa Cruz), Vimentin (1:1,000, Santa Cruz), SNAI1 (1:1,000, Santa Cruz), OCT4 (1:1,000, Cell Signaling Technology), LIN28A (1:1,000, Cell Signaling Technology), and GAPDH (1:1,000, Santa Cruz). Thereafter, the membranes were incubated with secondary antibodies conjugated with horseradish peroxidase (HRP) at room temperature for 1 h and carefully washed thrice with cold 1× PBS. The protein bands were detected with the enhanced chemiluminescence detection system (Thermo Fisher Scientific Inc., Waltham, MA, USA), and protein band densitometry was done using ImageJ software version 1.49 (https://imagej.nih.gov/ij/).

### Tumorsphere Formation and Self-Renewal Assay

5 × 10^4^ WT or shMED10 SW1738 and JMSU1 cells were seeded per well in ultra-low attachment 6-well plates (Corning, Corning, NY, USA) containing RPMI 1640 supplemented with Gibco™ B-27TM supplement (#17504044, Invitrogen, Carlsbad, CA, USA), 20 ng/ml basic fibroblast growth factor (bFGF; #13256029, Invitrogen), and 20 ng/ml epidermal growth factor (EGF; #PHG0311, Invitrogen). Cells were cultured at 37°C in a humidified 5% CO_2_ incubator for 6 days. Cultivated primary tumorspheres ≥ 100 µm were counted under inverted phase-contrast microscope, and then secondary tumorspheres were generated from the primary tumorspheres by dissociating them and reseeding the dissociated cells as per the primary tumorspheres, from single-cell suspension acquired using a sterile 22-G needle.

### Scratch-Wound Healing Migration Assay

We used the scratch wound-healing assay to assess cell migration. Briefly, WT or shMED10 bladder UC cells were seeded and allowed to grow in 6-well plates (Corning, Corning, NY, USA) containing complete growth media with 10% FBS. Media in wells were changed to low-serum (1% FBS) growth media when cells attained >98% confluence. The median axes of the monolayered adherent cells were scratched using sterile yellow pipette tips and carefully washed with low-serum media to rid detached cells. Cell migration based on scratch-wound healing was monitored over time, and images were captured at 0 and 12 h after denudation under a light microscope using a ×10 objective lens. Thereafter, the images were analyzed using the National Institutes of Health ImageJ software version 1.49 (https://imagej.nih.gov/ij/).

### Invasion Assay

Invasion assay was performed using the Corning^®^ BioCoat™ Matrigel^®^ invasion chambers with a 8.0−μm PET membrane in a two-24-well plate system (#354480, Corning, Corning, NY, USA). 1 × 10^5^ WT or shMED10 JMSU1 cells were seeded per well in plates and incubated at 4°C overnight. The upper chambers contained low-serum (2% FBS) media while the lower chamber contained 600 ;μl high-serum (20% FCS) media. After 48-h incubation, the non-invaded cells in the upper chamber were carefully wiped off with sterile cotton swabs, while the invaded cells that penetrated through the membrane were fixed with ethanol, stained with crystal violet solution, and counted under light microscope from six random fields of vision.

### Colony Formation Assay

2 × 10^4^ WT or shMED10 SW1738 and JMSU1 cells were seeded into 6-well culture plates (Corning Inc., Corning, NY, USA) and incubated for 13–15 days at 37°C. Cells were then washed three times with cold 1× PBS, fixed with ice-cold methanol, stained with 0.005% crystal violet, washed with 1× PBS, and dried at room temperature. The colonies formed were assessed and counted under microscope, as well as digitally using the National Institutes of Health ImageJ software version 1.49 (https://imagej.nih.gov/ij/). In each well, the colonies with diameter ≥ 100 μm were counted over six randomly selected fields in triplicate assays.

### Statistical Analysis

All data represent the mean ± standard deviation (SD) of assays performed at least 3 times in triplicates. The 2-sided Student’s *t* test was used for comparison between 2 groups, whereas one-way ANOVA with Tukey’s *post-hoc* test was used for comparison between 3 or more groups. Kaplan–Meier survival analyses aided comparison of survival rates between the control and test groups. All statistical analyses were performed using MS Excel (Microsoft Corporation. Microsoft Excel [Internet]. 2018. Available from: https://office.microsoft.com/excel) and GraphPad Prism version 8.0.0 for Windows (GraphPad Software, La Jolla, CA, USA). *p-*value < 0.05 was considered statistically significant.

## Results

### MED10 Is Aberrantly Expressed in Patients With BLCA, and This Has an Adverse Prognostic Implication

**Figure 1:**
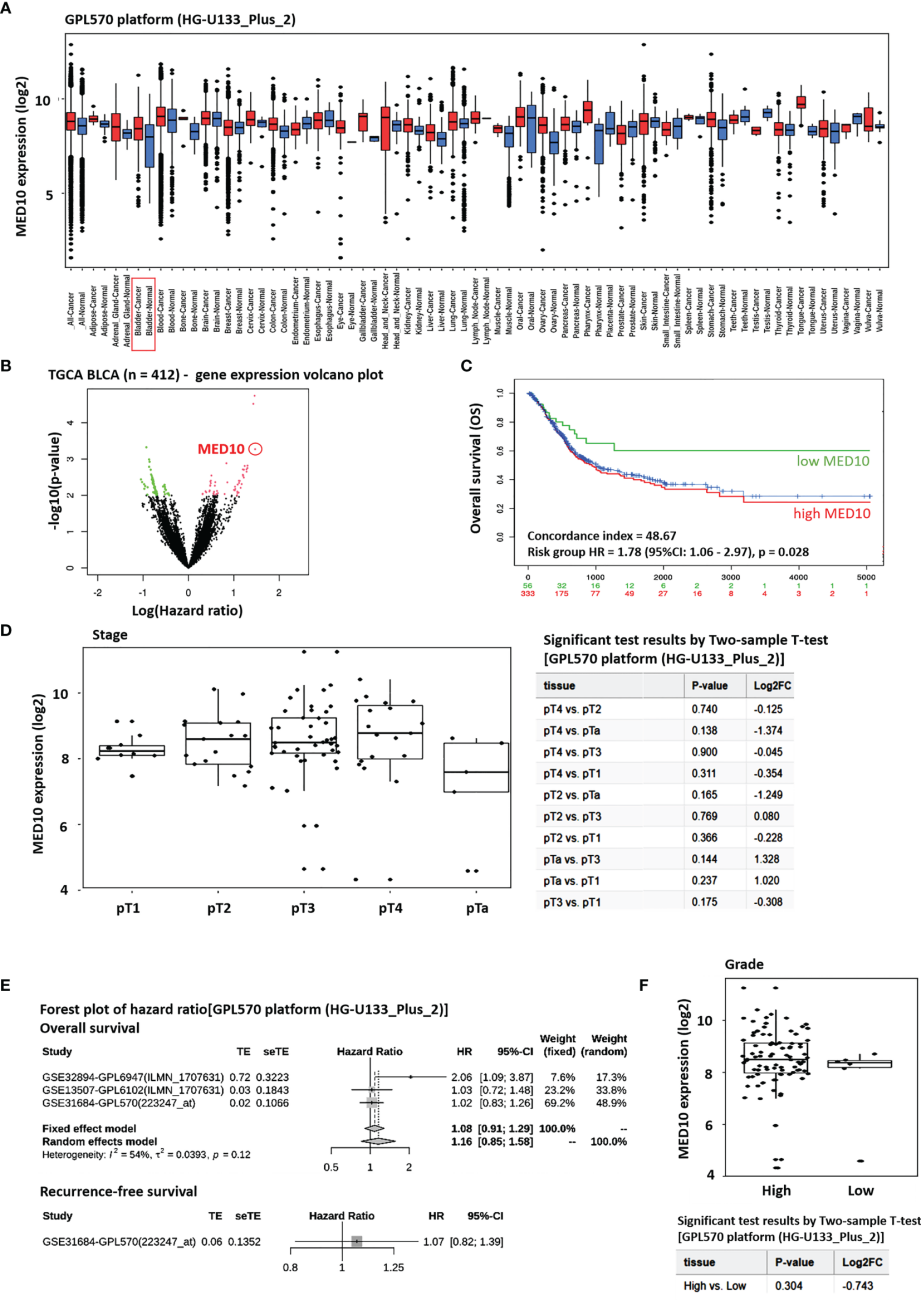
MED10 is aberrantly expressed in patients with BLCA, and this has an adverse prognostic implication. **(A)** Box plot of the tissue-wide differential gene expression profile across cancer experiments using the GPL570 platform (HG-U133_Plus_2)]. **(B)** Volcano plot of the hazard ratios of differentially expressed genes in the TGCA BLCA cohort. MED10 is indicated by the red circle. Volcano plot was plotted with log hazard ratio (HR) on the x-axis and p-value on the y-axis, using the Cox PH model. **(C)** Kaplan–Meier plot showing the effect of altered MED10 expression on the overall survival of patients in the BLCA cohort of the GPL570 platform. **(D)** Box and dot plots showing the association between MED10 expression and tumor stage in the GPL570 platform BLCA cohort *(left)*. Chart showing the 2-sample t-test results for **(D)** *(right)*. **(E)** Forest plot of hazard ratios showing the effect of MED10 on overall and recurrence-free survival on the GPL570 platform BLCA cohort. **(F)** Box and dot plots of the MED10 expression profile based on tumor grade in the GPL570 platform BLCA cohort.

Bioinformatics-aided analyses of the GPL570 platform [HG-U133_Plus_2] Affymetrix Human Genome U133 Plus 2.0 Array (n = 162,085) (https://www.ncbi.nlm.nih.gov/geo/query/acc.cgi?acc=GPL570) revealed that the expression of *MED10* transcripts was significantly higher in the BLCA patients than in their normal peers (1.66-fold, *p* = 0.002) (**Figure 1A**). This was corroborated by Cox PH modeling-based volcano plot visualization of differentially expressed genes in the TCGA BLCA cohort (n = 412), showing that the overexpression of the *med10* gene was associated with increased hazard ratio (3.53-fold, *p* < 0.001) (**Figure 1B**). Survival analyses of the TCGA BLCA cohort showed that compared with their high *MED10* counterparts (n = 333), patients with low *MED10* expression (n = 56) enjoyed an 18% to 27% survival advantage from days 1,000 to 5,000 (concordance index = 48.67; hazard ratio HR = 1.78 (95% CI: 1.06–2.97), *p* = 0.028) (**Figure 1C**). Using the GPL570 platform [HG-U133_Plus_2], we also demonstrated that increased *MED10* expression positively correlated with increased pathological tumor (pT) stage (**Figure 1D**). Our hazard ratio forest plot of BLCA/UC-relevant series in the GPL570 platform showed that high *MED10* expression favors increased likelihood of disease-specific death (HR = 1.16 (95% CI: 0.85–1.58) and recurrence (HR = 1.07 (95% CI: 0.82–1.39) (**Figure 1E**). More so, *MED10* expression was elevated in patients with high-grade BLCA compared to the low-grade peers, albeit statistically insignificant (**Figure 1F**). These data indicate that MED10 is aberrantly expressed in patients with BLCA and that this has an adverse prognostic implication.

### MED10 Expression Is Positively Correlated With Disease Progression, Immune Suppression, and Therapy Failure

**Figure 2:**
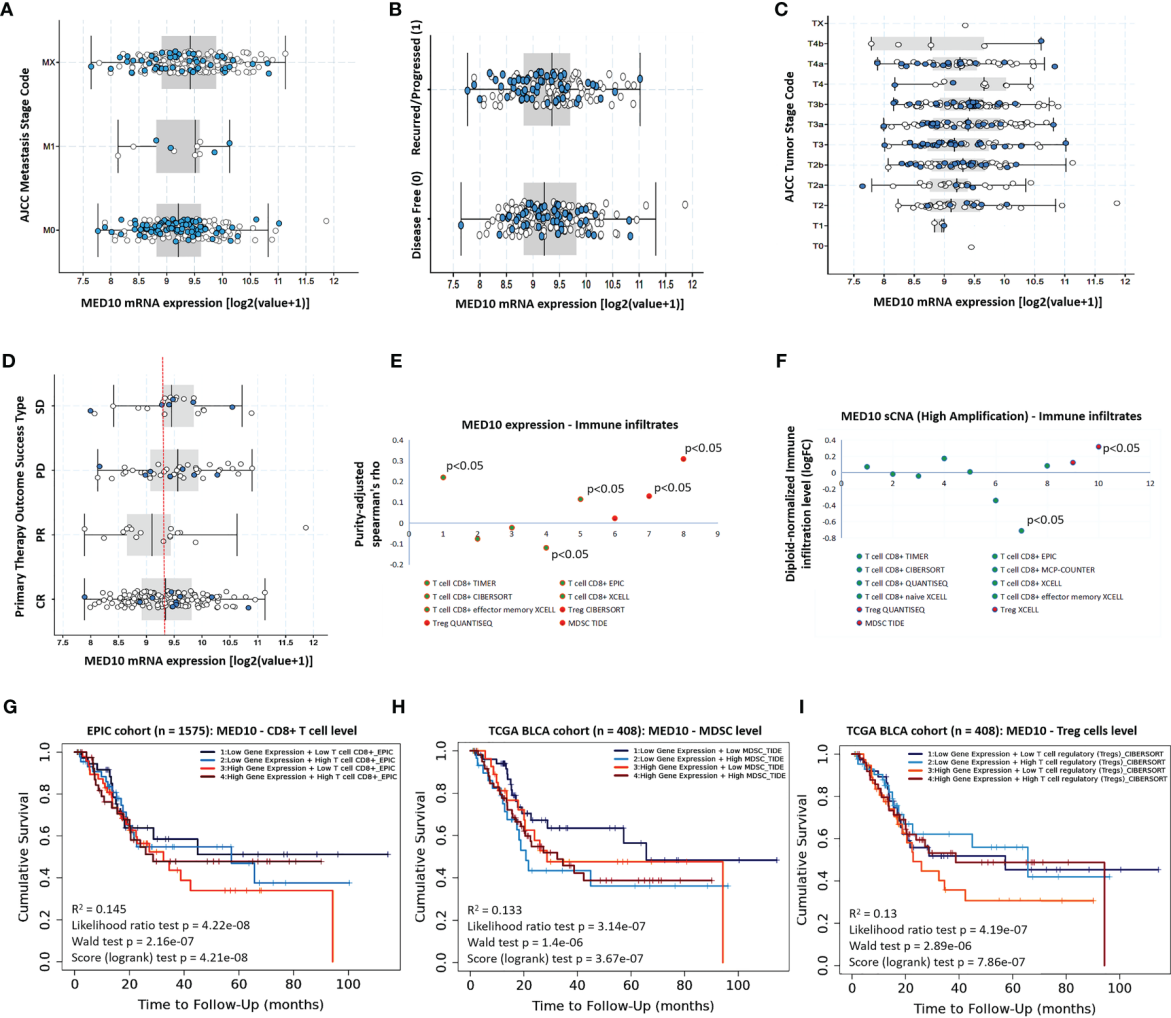
MED10 expression is positively correlated with disease progression, immune suppression, and therapy failure. Box plots showing the association between MED10 mRNA expression and **(A)** AJCC metastasis stage, **(B)** recurrence/progression or disease-free status of patients, **(C)** AJCC tumor stage, or **(D)** primary therapy outcome success type, in the TCGA BLCA cohort. **(E)** Dot plot of the tumor purity-adjust correlation between MED10 expression and level of tumor immune infiltrates. **(F)** Dot plot of the effect of high amplification of MED10 on the diploid-normalized immune cell infiltration level. Kaplan–Meier plot showing the effect of altered MED10 expression with or without altered **(G)** CD8+ T, **(H)** MDSC, or **(I)** Treg cell level on the cumulative survival.

Having demonstrated the aberrant expression of MED10 in patients with BLCA and its association with poor prognosis, we sort to gain further insight into the probable role of MED10 in disease progression and therapy response. Reanalyzing the TCGA BLCA cohort data, we demonstrated that compared to patients without metastasis (M0), *MED10* expression is upregulated in metastatic disease (M1) (**Figure 2A**). Similarly, *MED10* expression is higher in patients with recurred or progressed disease, compared with their disease-free counterparts (**Figure 2B**). Also, *MED10* transcript expression is positively correlated with increased American Joint Committee on Cancer (AJCC) tumor stage (T1 < T2 < T3 < T4) (**Figure 2C**). Interestingly, we also observed a higher median expression of MED10 in patients with progressive (PD) and stable (SD) disease, compared with their peers who had complete response/remission (CR), while the partial responders had the lowest *MED10* expression (PD > SD > CR > PR) (**Figure 2D**). More so, our systematical analysis of immune infiltrates in BLCA across diverse platforms using the TIMER 2.0 algorithm (19) showed that CD8+ cytotoxic T cells were largely inversely correlated with MED10 protein expression (Spearman’s rho_TIMER_ = 0.23, *p* < 0.05; Spearman’s rho_EPIC_ = -0.09, Spearman’s rho_CIBERSORT_ = -0.04, Spearman’s rho_XCELL_ = -0.15, *p* < 0.05), and infiltration of CD8+ effector memory T cells showed a mild positive correlation with MED10 expression (Spearman’s rho_XCELL_ = ~0.11, *p* < 0.05); however, MED10 expression was positively correlated with suppressors of immune response, namely, regulatory T cells, Treg (Spearman’s rho_CIBERSORT_ = 0.03; Spearman’s rho_QUANTISEQ_ = 0.13, *p* < 0.05) and myeloid-derived suppressor cells, and MDSC (Spearman’s rho_TIDE_ = 0.34, *p* < 0.05) (**Figure 2E**). Our correlative somatic copy number alteration (sCNA) analyses showed that compared to its ambivalent association with CD8+ T cells with Spearman’s rho ranging from -0.78 to 0.18, high amplification of *med10* gene is positively correlated with Treg infiltration (**Figure 2F**). Of clinical relevance, we also showed that patients with concurrent high *MED10* expression and low CD8+ T cell levels in the EPIC cohort (n = 1575) exhibited the worst cumulative survival rate (R^2^ = 0.15, *p* = 4.21e-08) (**Figure 2G**). Using the TCGA BLCA cohort (n = 408), we observed that patients bearing high *MED10* expression with or without high MDSC levels exhibited worse survival rates relative to those with concurrent low *MED10* and low MDSC levels (R^2^ = 0.13, *p* = 3.67e-07) (**Figure 2H**). Moreover, patients with concurrent high *MED10* and low Treg levels in the TCGA BLCA cohort (n = 408) exhibited the worst cumulative survival rate, compared to those with concomitantly high *MED10* and Treg levels or those with low *MED10* expression regardless of Treg level (R^2^ = 0.13, *p* = 7.86e-07) (**Figure 2I**). These data do indicate, at least in part, that MED10 expression is positively correlated with disease progression, immune suppression, and therapy failure.

### MED10 Is Functionally Co-Expressed With hsa-miR-590 but Is Inversely Associated With Tumor-Suppressor MicroRNAs

**Figure 3:**
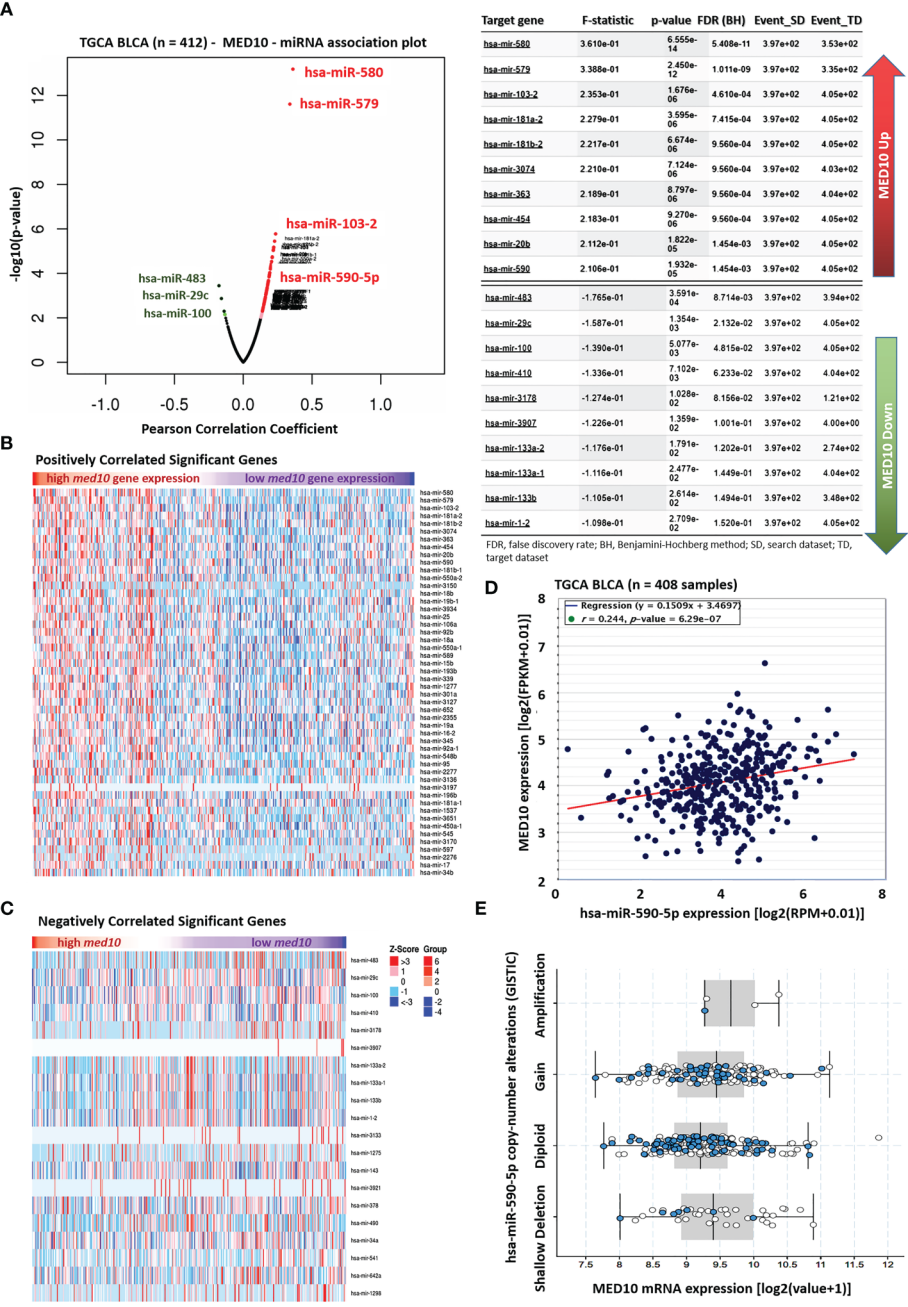
MED10 is functionally co-expressed with hsa-miR-590 but is inversely associated with tumor-suppressor microRNAs. **(A)** Association plot of the relationship between the expression of MED10 and miRs in the TCGA BLCA cohort *(left)*. Chart showing the 10 topmost miRs associated with upregulated or downregulated MED10 expression *(right)*. Heatmaps showing **(B)** positively and **(C)** negatively correlated significant miRs in the TCGA BLCA cohort. **(D)** Line and dot plots showing the correlation between MED10 and hsa-miR-590-5p expression levels in the TCGA BLCA cohort. FPKM, fragmented per kilobase of transcript per million; RPM, reads per million total/mapped reads. **(E)** Box and whiskers plot showing the association between MED10 mRNA expression and hsa-miR-590-5p copy number alterations.

Against the background that microRNAs (miRs) are critical regulators of gene expression, transcription, and translation (21, 22), seeking to gain some mechanistic insight into the oncogenic and immune-suppressing function of MED10 in BLCA/UC, we performed a MED10–miR association probe. Our MED10–miR association plot showed that MED10 was strongly associated with several miRs including a relatively unknown hsa-miR-590-5p (F-stat = 2.11, *p* = 1.93e-05) (**Figure 3A**). More so, using the TCGA BLCA cohort (n = 412), we demonstrated that upregulated expression of the *med10* gene is positively correlated with several oncogenic miRs (oncomiRs) including hsa-miR-590 (**Figure 3B**) but is inversely correlated with expression levels of tumor-suppressor miRs, including hsa-miR-483, hsa-miR-29c, hsa-miR-100, and hsa-miR-143 (**Figure 3C**). We also demonstrated a significant positive correlation between *MED10* and hsa-miR-590-5p transcript expression (r = 0.24, *p* = 6.29e-07) (**Figure 3D**). Consistent with this, using the GISTIC module (https://www.genepattern.org/modules/docs/GISTIC_2.0), we found that unlike in patients with diploid hsa-miR-590-5p, hsa-miR-590-5p copy number gain or amplification was associated with upregulated *MED10* transcript expression in patients with BLCA/UC (**Figure 3E**). These data indicate that MED10 is functionally co-expressed with hsa-miR-590 but is inversely associated with tumor-suppressor microRNAs.

### MED10 Interacts Directly With hsa-miR-590, a Modulator of Immune Infiltration and Survival

**Figure 4:**
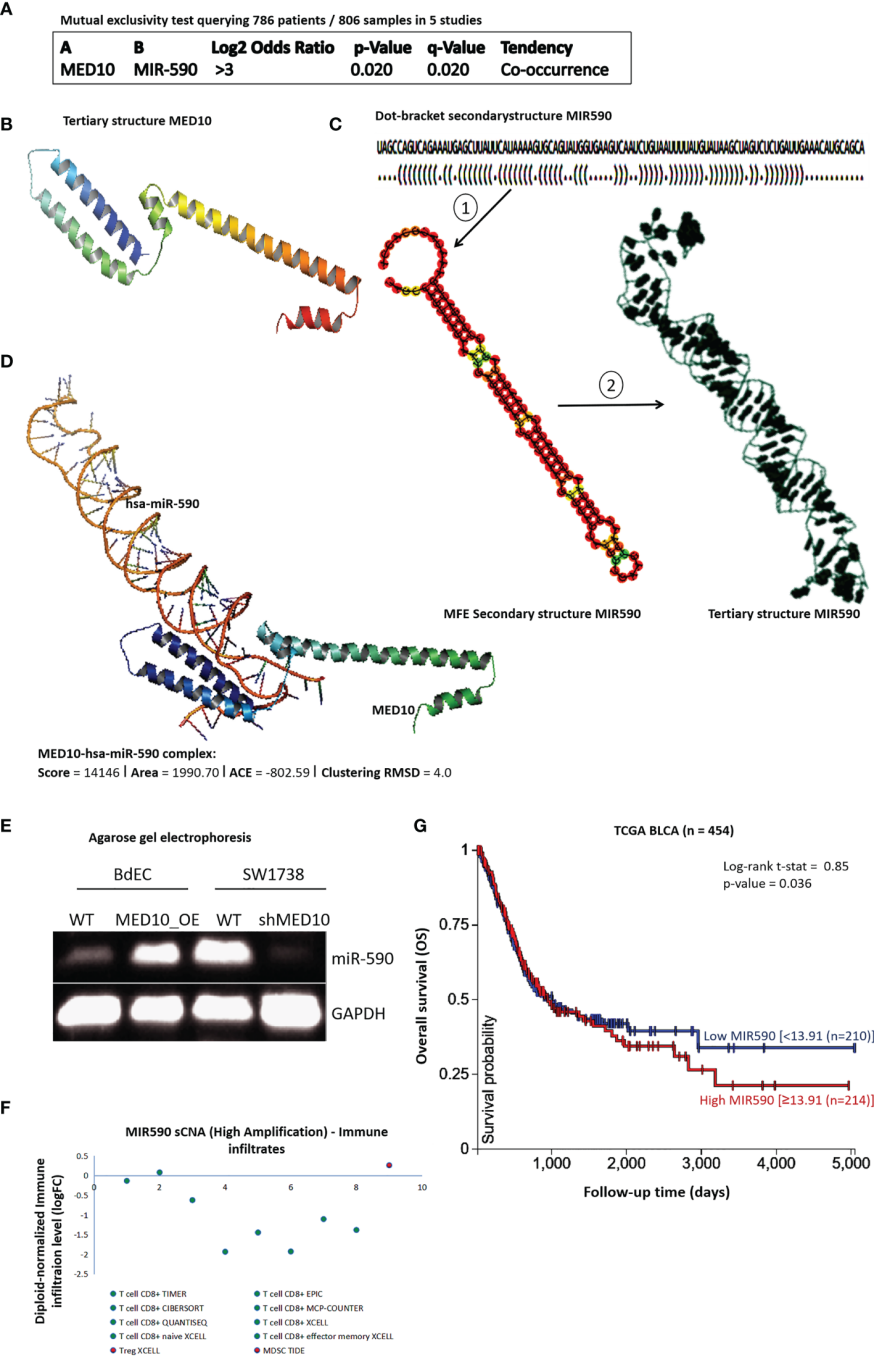
MED10 interacts directly with hsa-miR-590, a modulator of immune infiltration and survival. **(A)** Depiction of mutual exclusivity test data showing tendency and likelihood of MED10 and hsa-miR-590 to co-occur in pool of 5 BLCA/UC studies. **(B)** Sequence-derived 3D structure of MED10. **(C)** The optimal secondary structure in dot-bracket notation with a minimum free energy (MFE) of −31.20 kcal/mol *(upper)*. The RNAfold-generated MFE secondary folding structure pattern of hsa-miR-590 *(lower left)*. The hsa-miR-590 3D structure generated based on the secondary structure in dot-bracket notation *(lower right)*. **(D)** Molecular docking showing the direct interaction between MED10 and hsa-miR-590. The 3D transformation data, consisting of three rotational angles (2.42°, 0.38°, 120.70°) and three translational parameters (−1.18, −53.27, −102.28) applied on the ligand molecule, hsa-miR-590. **(E)** Representative agarose gel electrophoresis image of the effect of altered MED10 protein expression on the expression level of hsa-miR-590 in BdEC and SW1738 cells. GAPDH served as loading control. WT, wild type. **(F)** Dot plot showing the effect of high amplification of hsa-miR-590 on the immune cell infiltration levels. **(G)** Kaplan–Meier plot of the effect of altered hsa-miR-590 expression in the TCGA BLCA cohort.

Since co-expression does not necessarily translate into molecular interaction, having shown that MED10 is functionally co-expressed with hsa-miR-590, we probed for a probable interaction between MED10 and hsa-miR-590. Using the mutual exclusivity test for MED10 and hsa-miR-590 in a pooled BLCA cohort (n = 786 patients, 806 samples) consisting of bladder cancer (MSK/TCGA, 2020 n = 476), bladder cancer (MSKCC, Eur Urol 2014 n = 109), bladder urothelial carcinoma (BGI, Nat Genet 2013 n = 99), bladder urothelial carcinoma (DFCI.MSKCC, Cancer Discov 2014 n = 50), and urothelial carcinoma (Cornell/Trento, Nat Gen 2016 n = 72), we confirmed the spatiotemporal association between the dyad (co-occurrence: log2 odds ratio >3, *p* = 0.02) (**Figure 4A**). To better understand the MED10/hsa-miR-590-5p relationship and for visualization of our hypothesized molecular interaction between MED10 and hsa-miR-590-5p in BLCA/UC cells, we employed a bioinformatics approach to generate the tertiary (3D) structure of MED10 based on sequence homology modeling (NCBI Reference Sequence: NP_115662.2) (https://www.ncbi.nlm.nih.gov/protein/NP_115662.2; **Figure 4B**). We also generated the 3D structure of hsa-miR-590 from its sequence (NCBI Reference Sequence: NR_030321.1)-derived “brackets and dots” linear structure and the minimum free energy (MFE) secondary (2D) structure (**Figure 4C**). Using the Schrödinger PyMOL 2.5 molecular interaction and visualization software (https://pymol.org/2/), we observed a high interaction propensity, broad conservation, and good complementarity between the 5′ end of hsa-miR-590 and the C-terminal RNA-binding motif of MED10, demonstrated by a shape complementarity/docking score of 14,146, atomic contact energy (ACE) of −802.59 kcal/mol, approximate MED10/hsa-miR-590-5p complex interface area of 1,990.70 Å^2^, root-mean-square deviation (RMSD) of atomic positions of 4.0 Å, and a *p*-value of 3.11e−04, with the 3D transformation data, consisting of three rotational angles (2.42°, 0.38°, 120.70°) and three translational parameters (−1.18, −53.27, −102.28) applied on the ligand molecule, hsa-miR-590 (**Figure 4D**). Furthermore, to confirm the functional and/or modulatory nature of the demonstrated MED10/hsa-miR-590-5p interaction, using agarose gel electrophoresis, we showed that the ectopic expression of MED10 in normal human primary bladder epithelial BdEC cells significantly upregulated the expression of hsa-miR-590, compared to the WT cells; more so, upon silencing MED10 (shMED10) in metastatic human bladder transition cell carcinoma SW1738 cells, hsa-miR-590 expression was markedly suppressed (**Figure 4E**). These findings indicate that MED10 interacts directly with and upregulates hsa-miR-590 expression in BLCA/UC cells. For functional insights, using the TIMER 2.0 algorithm (23), our correlative sCNA analyses showed that akin to MED10, high amplification of hsa-miR-590 was largely correlated with suppressed CD8+ T cell infiltration level but is positively correlated with Treg infiltration (Spearman rho_XCELL_ = 0.31) (**Figure 4F**). Moreover, survival analysis of the TCGA BLCA cohort (n = 454) showed that high hsa-miR-590 expression conferred survival disadvantage compared to low expression (t-stat = 0.85, *p* = 0.04) (**Figure 4G**). These data indicate, at least in part, that MED10 interacts directly with hsa-miR-590, a modulator of immune infiltration and survival.

### Targeting MED10 Elicits Downregulation of hsa-miR-590-5p Expression Preferentially in Metastatic, Transitional Cell (Urothelial) Carcinoma Cells

**Figure 5:**
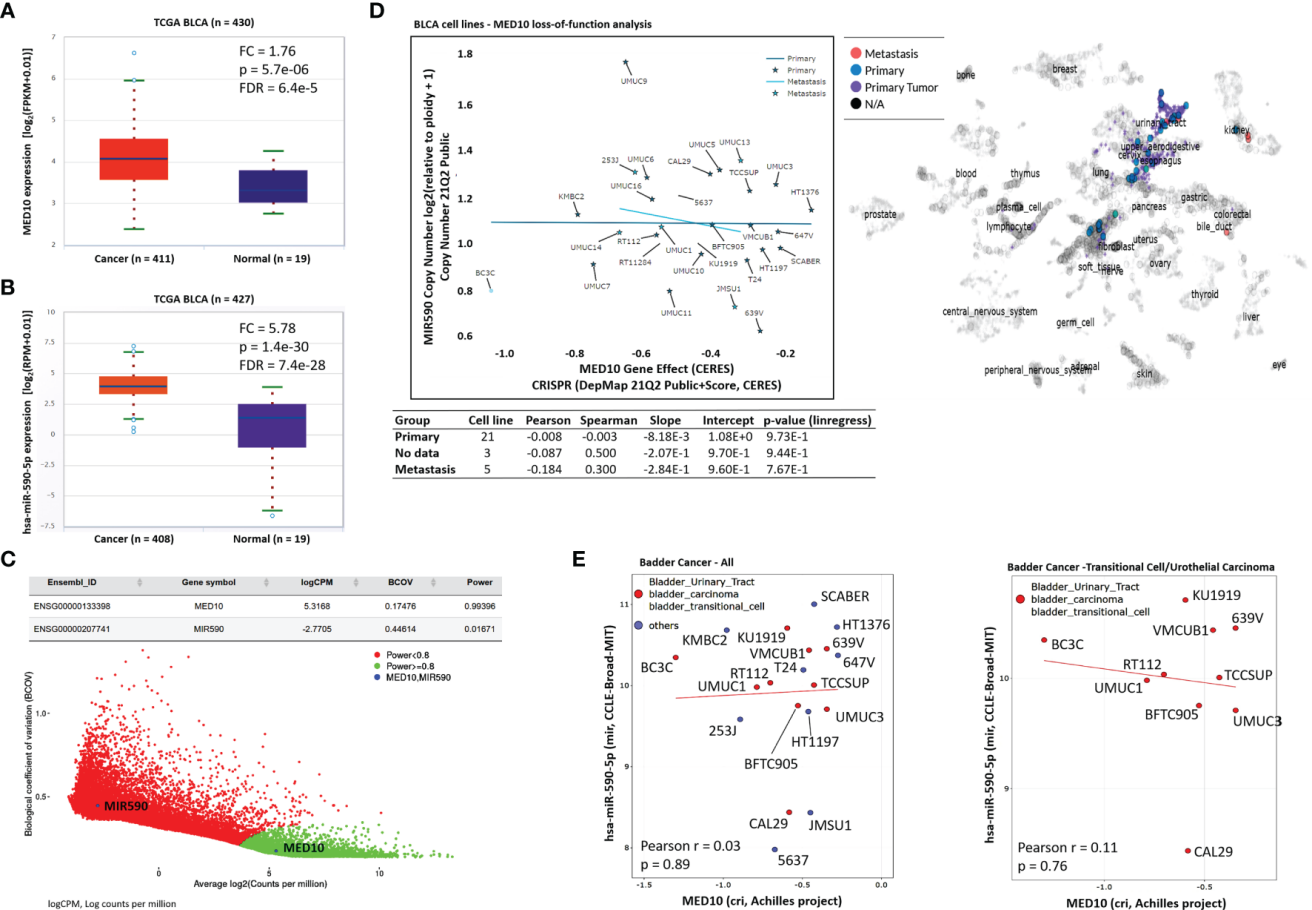
Targeting MED10 elicits downregulation of hsa-miR-590-5p expression preferentially in metastatic, transitional cell (urothelial) carcinoma cells. Box and whisker plots of the differential expression of **(A)** MED10 or **(B)** hsa-miR-590-5p in cancer and normal samples from the TCGA BLCA cohort. **(C)** Scatter-plot of the biological coefficient of variation (BCOV) against the average abundance of MED10 and MIR590 in the GSE81157 aggressive bladder cancer cohort. **(D)** Graphical representation of the effect of MED10 loss of function on hsa-miR-590 copy number in 29 metastatic or primary BLCA cell lines from the 21Q2 Public cohort *(left)*. Chart showing the spatial distribution of the BLCA cell lines according to metastasis status *(right)*. Visualization of the effect of MED10 knockout on hsa-miR-590-5p expression in **(E)** mixed pool of BLCA cell lines *(left)*, or purely bladder transitional/UC cell lines *(right)*.

Reaffirming previous data, we demonstrated similarity in the cancer-normal differential expression profile of *MED10* (fold change, FC = 1.76, *p* = 5.7e-06) and hsa-miR-590-5p (FC = 5.78, *p* = 1.4e-30) in the TCGA BLCA cohort (**Figures 5A, B** **)**. To rule out multifactorial inter-sample inconsistencies/variation and establish replicability of the observed expression profile, our gene detectability power analysis of the GSE81157 aggressive BLCA cohort (n = 9) showed that variation in the true abundance of *MED10* (biological coefficient of variation, BCOV = 0.17, power = 0.99) or hsa-miR-590 (BCOV = 0.45, power = 0.02) between replicate RNA samples was apparently insignificant (**Figure 5C**). Bioinformatics-aided evaluation of CRISPR-induced loss of MED10 function (criMED10) in 29 BLCA cell lines showed that increased suppression of *med10* elicited increased downregulation of hsa-miR-590 copy number in metastatic cell lines 253J, UMUC1, UMUC13, UMUC14, and JMSU1 (Spearman r = 0.30) compared with the apparent non-effect in primary tumor cell lines (Spearman r = -0.003) (**Figure 5D**). Furthermore, in a non-stratified CCLE–Broad–MIT pool of BLCA cells, criMED10 had no apparent effect (Pearson r = 0.03); however, upon extraction and probe of only transitional cell carcinoma/UC cells, we found that criMED10 elicits suppressed expression of hsa-miR-590-5p in the BC3C, UMUC1, RT112, TCCSUP, BFTC905, UMUC3, VMCUB1, KU1919, 639V, and CAL29 UC cell lines (**Figure 5E**). These data indicate that targeting MED10 elicits downregulation of hsa-miR-590-5p expression preferentially in metastatic, transitional cell (urothelial) carcinoma cells.

### shRNA-Mediated Targeting of MED10 in Bladder Urothelial Carcinoma Cells Significantly Attenuates Their Oncogenicity and Metastatic and Cancer Stemness Phenotypes

**Figure 6:**
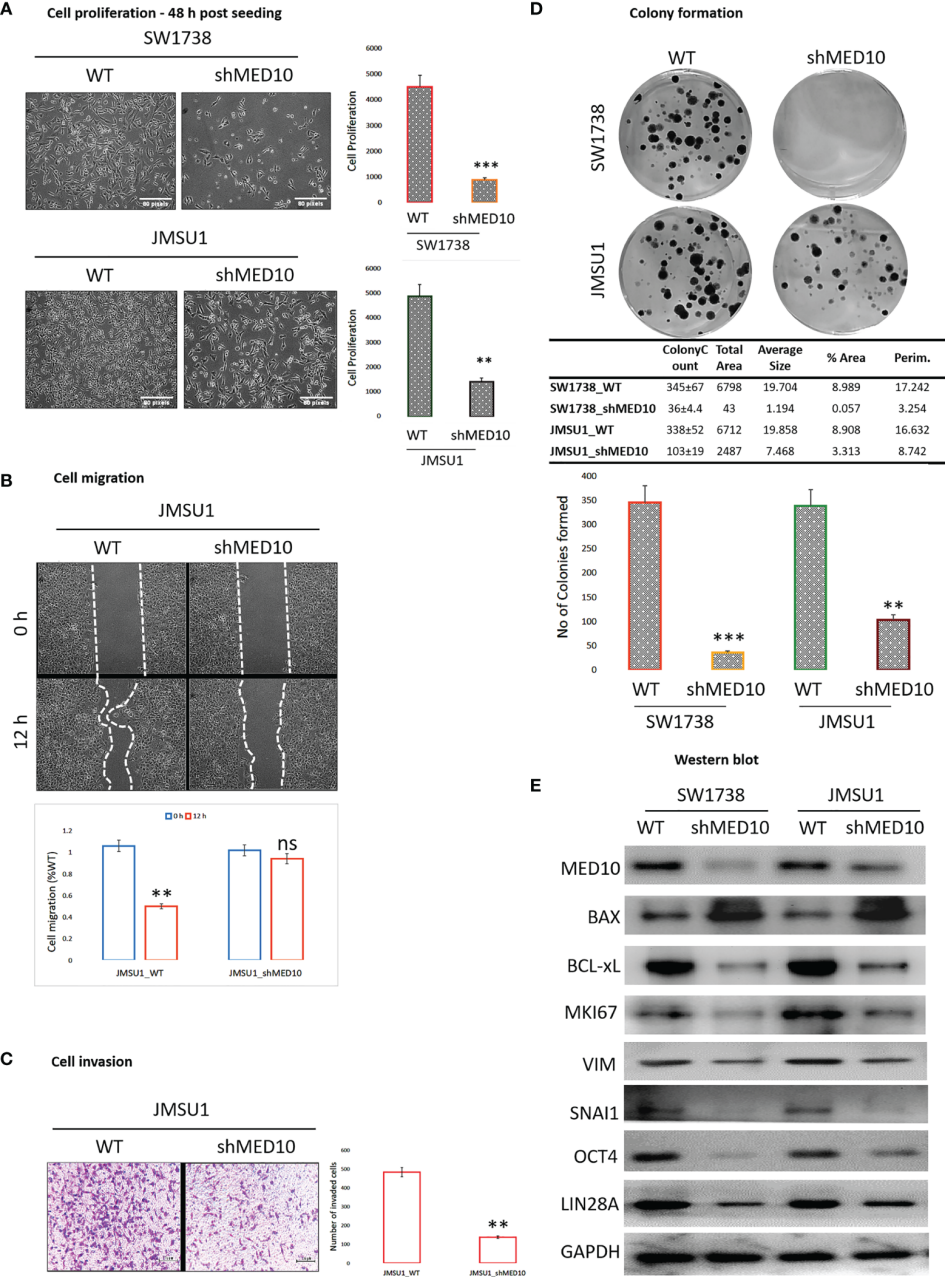
shRNA-mediated targeting of MED10 in bladder urothelial carcinoma cells significantly attenuate their oncogenicity and metastatic phenotype. Photomicrographs and histograms showing the effect of knocking down MED10 on the **(A)** proliferation, ***(*B*)*** migration, **(C)** invasion, and **(D)** colony formation of SW1738 and JMSU1 cells. **(E)** Representative Western blot images showing the effect of shMED10 on the expression of MED10, BAX, BCL-xL, VIM, SNAI1, OCT4, or LIN28A protein expression level in SW1738 and JMSU1 cells. GAPDH serve as loading control. ns, not significant; **p < 0.01; ***p < 0.001.

**Figure 7:**
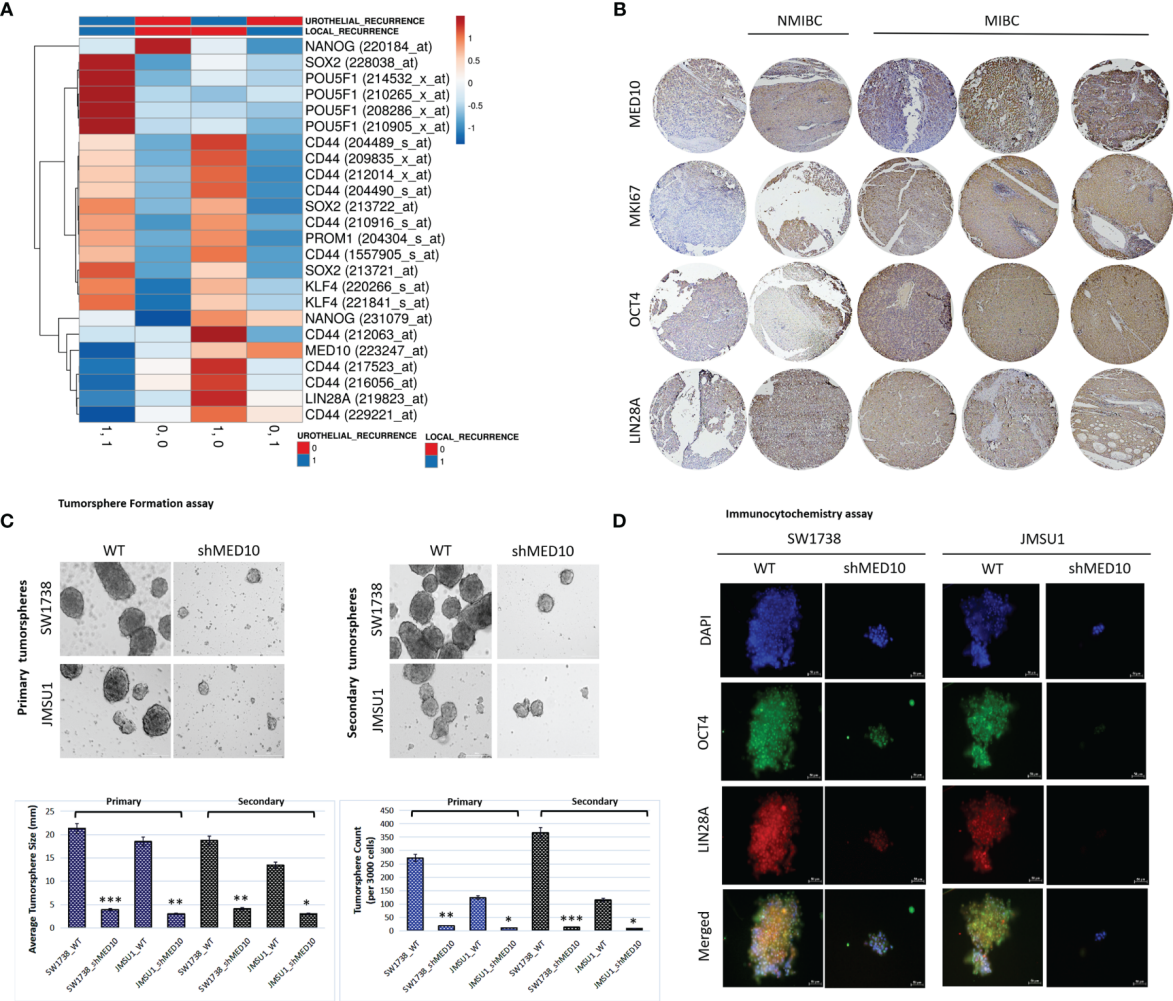
shRNA-mediated targeting of MED10 in bladder urothelial carcinoma cells significantly attenuate their cancer stemness phenotype. **(A)** Heatmap showing the correlation between the expression of MED10, and stemness markers NANOG, SOX2, POU5F1/OCT4, CD44, PROM1/CD133, KLF4, and LIN28A in patients with urothelial recurrence or local recurrence from the AFFY_HG_U133_PLUS_2, GSE31684 cohort. Columns with similar annotations are collapsed by taking mean inside each group. Rows are centered; unit variance scaling is applied to rows. Both rows and columns are clustered using correlation distance and average linkage. 24 rows, 4 columns. **(B)** Immunohistochemistry photomicrographs of the differential expression of MED10, MKI67, OCT4, and LIN28A in benign, T1, T2, T3/4, or M1 tissue samples from the TMU-SHH UC cohort. Scale bar 200 μm. **(C)** Photomicrographs and histograms showing the effect of shMED10 on the formation of primary or secondary tumorspheres from SW1738 and JMSU1 cells. **(D)** Representative immunofluorescence images showing the effect of knocking down MED10 on the nuclear translocation and co-localization of OCT4A and LIN28A. DAPI served as nuclear marker. Scale bar 50 μm. *p < 0.05; **p < 0.01; ***p < 0.001.

Having shown that targeting MED10 elicits downregulation of hsa-miR-590-5p expression preferentially in metastatic, transitional cell (urothelial) carcinoma cells, for functional characterization of the effect of altered MED10/hsa-miR-590-5p signaling in UC cells, we performed several functional assays. We observed that shMED10 significantly suppressed the proliferation of SW1738 (4.47-fold, *p* < 0.001) and JMSU1 (4.88-fold, *p* < 0.01) cells (**Figure 6A**). Results of our migration assays demonstrate that shMED10 markedly attenuated the migration of JMSU1 cells (2.11-fold, *p* < 0.01) (**Figure 6B**). shMED10 also significantly inhibited the invasive capability of JMSU1 cells (4.35-fold, *p* < 0.01) (**Figure 6C**). Similarly, we demonstrated that shMED10 profoundly suppressed the ability of the SW1738 (9.58-fold, *p* < 0.001) and JMSU1 (3.28-fold, *p* < 0.01) cells to form colonies (**Figure 6D**). In parallel assays, we found that shMED10 significantly downregulated the expression levels of MED10, anti-apoptosis BCL-xL, proliferation marker MKI67/Ki67, biomarkers of metastasis VIM and SNAI1, and stemness/pluripotency markers OCT4 and LIN28A, while concomitantly upregulating pro-apoptosis BAX protein in both the SW1738 and JMSU1 cells (**Figure 6E**). Because of the association of onco-aggression and disease recurrence with cancer stemness (21), we assessed for probable effects of altered MED10/hsa-miR-590 signaling on cancer stem cell activities in UC cells. Reanalysis of the AFFY_HG_U133_PLUS_2, GSE31684 UC cohort (n = 93) showed that MED10 is co-overexpressed with stemness markers CD44, SOX2, PROM1/CD133, KLF4, NANOG, and LIN28A in patients with urothelial recurrence and is concomitantly upregulated with NANOG and CD44 in patients with local recurrence, but profoundly suppressed, akin to the other stemness/pluripotency markers, in patients with non-recurrent disease (**Figure 7A**). Interestingly, using our in-house bladder UC tissue samples (n = 79), we showed that compared with the benign tissue samples, MED10 expression, alongside Ki67, OCT4, and LIN28A, is disease progression from non-muscle invasive bladder cancer (NMIBC: pT1) to muscle-invasive bladder cancer (MIBC: pT2–pT4) and more so in patients with distant metastatic disease (M1) (benign < T1 < T2 < T3/4 < M1) (**Figure 7B**). Reminiscent of the loss of self-renewal capability, we demonstrated that shMED10 significantly suppressed the ability of the SW1738 and JMSU1 cells to form primary and subsequently secondary tumorspheres, quantitatively and qualitatively (**Figure 7C**), with concomitant inhibition of the nuclear translocation and co-localization of the pluripotency/stemness markers OCT4 and LIN28A (**Figure 7D**). This is further corroborated by our biological function gene ontology (GO) enrichment analysis showing that MED10 networks with hsa-miR-590, KLF4, SOX2, OCT4/POU5F1, LIN28A, and NANOG and is implicated in “stem cell population maintenance” (GO:0019827: FDR-adjusted *p* = 5.0e-10), “somatic stem cell population maintenance” (GO:0035019: FDR-adjusted *p* = 7.2e-09), “maintenance of cell number” (GO:0098727: FDR-adjusted *p* = 5.0e-10), and “cell fate commitment” (GO:0045165: FDR-adjusted *p* = 3.3e-04) (**Supplementary Figure 1**). These data demonstrate that targeting MED10 in bladder UC cells significantly attenuates their oncogenicity and metastatic and cancer stemness phenotypes.

## Discussion

Bladder UC, one of the most frequently diagnosed cancers globally, is increasingly characterized by early metastasization, unabated disease recurrence after initial response, and dismal prognosis in spite of diagnostic and therapeutic advances. This necessitates the identification and characterization of probable molecular mechanisms underlying its progression as a prelude for discovery of novel actionable diagnostic and prognostic biomarkers, and development of new efficacious therapeutic strategies for patients with UC.

The Mediator complex plays an essential role in the transduction of signals from enhancer region-bond activators of transcription to promoter site-associated RNA Pol II basal transcription machinery, to activate or repress transcription factors at distant genomic regions (10, 11, 24). This alteration of transcription factor activity with associated alterations in gene expression is characteristic of human health and diseases, including BLCA (25–27). In the present study, we provide preclinical evidence that MED10, a regulator of transcription, is aberrantly expressed in patients with BLCA and this has an adverse prognostic implication. This is consistent with accruing evidence of the tumor-promoting roles of components of the multi-protein Mediator complex, including reports that Mediator subunit CDK19 is specifically expressed in prostate cancer, with upregulated expression being associated with disease progression, as observed in patients with metastatic and castration-resistant disease (28). More so, several reports indicate that other subunits of the Mediator complex, which are actively involved in regulating estrogen and androgen receptor gene expression, are altered in some endocrine malignancies, such as prostate and breast cancer (19, 20, 29). We also show, for the first time to the best of our knowledge, that MED10 expression is positively correlated with disease progression, immune suppression, and therapy failure. This is in part corroborated by reports that the upregulation of MED28 expression by FOXD3-AS1, with concomitant suppression of miR-127-3p, promoted non-small cell lung cancer (NSCLC) cell proliferation and invasion, *in vitro*, and enhanced xenograft tumor growth (30). Considering the systemic nature of cancer, and the significant alteration observed in the global immune landscape of patients with refractory, metastatic, and/or recurrent cancer, our data showing that elevated *MED10* expression is associated with low levels of tumor-infiltrating CD8+ T cells and high MDSC and Treg levels is of therapeutic relevance, especially as the peripheral immune system is an essential driver of effective innate and treatment-induced anticancer immune responses (31). Tregs are required to maintain host immune tolerance and often cross talk with conventional T cell signaling, including those from the principal mediator of anticancer immunity, CD8+ T cells; however, Foxp3+ Tregs suppress anticancer immunity, facilitate evasion of immunosurveillance, hamper efficient anticancer immune response, and confer resistance to therapy (31, 32). Consistent with accruing evidence that sensitivity to anticancer therapy is favored by the pooling and activity of activated CD8+ T cells within the tumor microenvironment, with associated enhanced CD8+ T cell-based immune response, we posit that MED10 induces an immunosuppressive tumor microenvironment, by upregulating Treg and/or MDSC activity, dysregulating associated immune regulatory molecules, hampering lymphocyte homing, and depleting metabolites required for CD8+ T cell differentiation and function, all of which are implicated in reduced sensitivity or resistance to anticancer therapy (32 – 33). Thus, targeting MED10 may represent an alternative approach to inducing effective immune responses and/or re-invigorating preexisting anticancer immune responses.

Furthermore, against the background of the just emerging role of the Mediator complex in the regulation of non-coding RNAs, we demonstrated that MED10 is co-expressed with hsa-miR-590 and interacts directly with hsa-miR-590 but is inversely associated with tumor-suppressor microRNAs and that this interaction modulates immune infiltration and survival. The broadly documented differential expression of miRs in malignancies is increasingly considered a tenet of cancer initiation, progression, and therapy response (34). Corollary to our finding, it was recently reported that increased expression of Mediator subunit MED1 induces upregulation of the miR-191/425 cluster (namely, miR-100-5p, miR-191-5p, miR-193b-3p, miR-205-5p, miR-326, miR-422a and miR-425-5p), in breast cancer, and that this promotes cell proliferation and migration (35). More so, concordant with our finding that high hsa-miR-590 expression conferred survival disadvantage compared to low expression, a recent study based on single-sample gene-set enrichment analysis (ssGSEA) and least absolute shrinkage and selection operator (LASSO) Cox regression modeling suggested that certain miRs are significantly associated with immune-related response and pathways that are critical for initiation and progression of head and neck squamous cell carcinoma (HNSCC) (36). In addition, the expression of these immune-related miRs was shown to be strongly correlated with immune cell infiltration and expression of immune checkpoints, thus implicating probable immunosuppressive microenvironment in patients’ poor prognoses (36).

Despite common knowledge that nearly all bladder UC-specific deaths follow from metastatic disease, the genomic bio-drivers of disease progression and metastatic recurrence remain poorly understood. Interestingly, and of clinical relevance especially in the context of patient stratification for precision medicine, we demonstrated that targeting MED10 elicits downregulation of hsa-miR-590-5p expression preferentially in metastatic, transitional cell (urothelial) carcinoma cells and significantly attenuates their oncogenicity and metastatic and cancer stemness phenotypes. We posit that this preferential downregulation of the hsa-miR-590-5p sequel to MED10 inhibition is suggestive of a putative role for altered MED10/hsa-miR-590-5p signaling in the management of patients with metastatic or recurrent bladder UC, and in the light of other data present herein, we speculate that MED10 is a promising molecular candidate for urothelial cancer immunotherapy. Howbeit thematically different, the team of Lewis Chodosh in a contextually analogous study (37) demonstrated that certain genes, namely, MYLK, PEAK1, SLC2A4RG, EVC2, XIRP2, PALB2, and ESR1, and pathways such as WNT/β-catenin, PI3K/mTOR, CDK/RB, and cAMP/PKA are mutated or exhibit sCNA preferentially in metastases, compared with paired primary tumors. Pooled together with ours, these findings provide some genomic bases for developing efficacious targeted or immune-based therapy for metastatic disease. Our finding is also reminiscent of the oncogene addiction concept (38); inferring from our data, it is rationally conceivable that metastatic/recurrent bladder UC are addicted to MED10/hsa-miR-590-5p signaling, thus representing a therapeutic Achilles’ heel that makes these metastatic UC cells particularly susceptible or sensitive to inhibition of MED10 singly.

Moreover, concurring that “cancer stem cells are the leading power behind tumor growth, with the ability of self-renewal, metastasis, and resistance to conventional chemotherapy” (39), our data showing that MED10 is co-overexpressed with stemness markers CD44, SOX2, PROM1/CD133, KLF4, NANOG, and LIN28A in patients with urothelial recurrence and concomitantly upregulated with NANOG and CD44 in patients with local recurrence, but profoundly suppressed, akin to the other stemness/pluripotency markers, in patients with non-recurrent disease, are also clinically relevant. This finding is also corroborated, in part, by studies showing that the master pluripotency transcription factors Oct4, Sox2, and Nanog bind to cis-acting enhancers and recruit Mediator to activate most of the pluripotent embryonic stem cell (ESC) gene expression program (40). It thus may be inferred that the reduced expression of MED10 (and by inference, pluripotency factors) “cause preferential loss of expression of super-enhancer-associated genes” which are essential for maintaining “cancer cell identity” and promoting oncogene transcription (40, 41). Once again, this bio-event is suggestive of how MED10-induced or -mediated alteration in gene (stemness, oncogenic) expression may be exploited for development of an efficacious anti-metastasis, anti-recurrence therapeutic strategy in bladder UC clinics.

## Conclusions

These data provide preclinical evidence that dysregulated MED10/MIR590 signaling drives onco-aggression, disease progression, and recurrence of bladder UC and that this oncogenic signal is therapeutically actionable for repressing the metastatic/recurrent phenotypes, enhancing therapy response, and shutting down stemness-driven disease progression and relapse in patients with BLCA/UC.

## Data Availability Statement

The original contributions presented in the study are included in the article/**Supplementary Material**. Further inquiries can be directed to the corresponding authors.

## Ethics Statement

A total of 79 archived bladder urothelial carcinoma (UC) specimens were obtained from the Pathology Department of Taipei Medical University–Shuang Ho Hospital. These samples, originally collected for clinical diagnostics, were fully de−identified. The Institutional Review Board reviewed and addressed a related non−compliance (NC) event, confirming that appropriate corrective actions were taken in line with ethical and regulatory standards.

## Author Contributions

OAB, C-CW—study conception and design, collection and assembly of data, data analysis and interpretation, and manuscript writing. Y-HW, S-WH, W-LW, C-TY, C-CW—data analysis and interpretation. OAB, C-CW—provision of resources and administrative oversight. All authors contributed to the article and approved the submitted version.

## Funding

This study was also supported by grants from the Teh-Tzer Study Group for Human Medical Research Foundation (TMRF) [A1091044] to OAB and grants from the Ministry of Science and Technology (MOST) to C-CW [MOST-110-2314-B-038-038] and OAB [MOST-110-2314-B-038-035].

## Conflict of Interest

The authors declare that the research was conducted in the absence of any commercial or financial relationships that could be construed as a potential conflict of interest.

## Correction note

A correction has been made to this article. Details can be found at: 10.3389/fonc.2025.1667245.

## Publisher’s Note

All claims expressed in this article are solely those of the authors and do not necessarily represent those of their affiliated organizations, or those of the publisher, the editors and the reviewers. Any product that may be evaluated in this article, or claim that may be made by its manufacturer, is not guaranteed or endorsed by the publisher.

## Supplementary Material

Supplementary Figure 1 The MED10/MIR590 signaling plays a critical role in stem cell population maintenance and cell fate determination. GO term enrichment was based on biological function. GO, gene ontology.
