# Correction: MED10 drives the oncogenicity and refractory phenotype of bladder urothelial carcinoma through the upregulation of hsa-miR-590

**DOI:** 10.3389/fonc.2025.1667245

**Published:** 2025-08-28

**Authors:** Chia-Chang Wu, Yuan-Hung Wang, Su-Wei Hu, Wen-Ling Wu, Chi-Tai Yeh, Oluwaseun Adebayo Bamodu

**Affiliations:** ^1^ Department of Urology, Shuang Ho Hospital, Taipei Medical University, New Taipei City, Taiwan; ^2^ Taipei Medical University (TMU) Research Center of Urology and Kidney, Taipei Medical University, Taipei City, Taiwan; ^3^ Department of Urology, School of Medicine, College of Medicine, Taipei Medical University, Taipei City, Taiwan; ^4^ Graduate Institute of Clinical Medicine, College of Medicine, Taipei Medical University, Taipei, Taiwan; ^5^ Department of Medical Research, Shuang Ho Hospital, Taipei Medical University, New Taipei City, Taiwan; ^6^ Department of Medical Laboratory Science and Biotechnology, Yuanpei University of Medical Technology, Hsinchu City, Taiwan; ^7^ Department of Hematology and Oncology, Cancer Center, Shuang Ho Hospital, Taipei Medical University, New Taipei City, Taiwan

**Keywords:** bladder urothelial carcinoma, MED10, hsa-miR-590, metastasis, cancer stemness, disease progression, recurrence, therapy failure

In the published article, there was an error. The published article did not fully disclose the source of the human tissue samples or provide complete information about the relevant ethical approvals, including a noted non−compliance (NC) event.

A correction has been made to the section **Materials and Methods,** *Bladder Cancer Tissue Samples*, paragraph 1:

“Bladder Cancer Tissue Samples

A total of 79 archived tissue specimens diagnosed with bladder urothelial carcinoma (UC) were retrieved from the Department of Pathology at Taipei Medical University–Shuang Ho Hospital. These samples were originally collected for routine clinical diagnostic purposes and subsequently preserved in the hospital’s pathology archives. As the research was retrospective and involved the analysis of previously collected, fully de−identified specimens, the Institutional Review Board (IRB) of Taipei Medical University reviewed the protocol. It addressed a related non−compliance (NC) event. Upon review, the IRB concluded that the non−compliance of this study was appropriately managed and that corrective measures were implemented in accordance with ethical and regulatory standards governing human subject research.”

The ethics statement was erroneously given as “The studies involving human participants were reviewed and approved by the Joint Institutional Review Board (JIRB) of Taipei Medical University. The procurement of the samples was strictly adherent to the approved IRB (No. N202102034) issued by the JIRB. Informed consent was waived because of the retrospective nature of the study. Written informed consent for participation was not required for this study in accordance with the national legislation and the institutional requirements.”

The correct ethics statement is “A total of 79 archived bladder urothelial carcinoma (UC) specimens were obtained from the Pathology Department of Taipei Medical University–Shuang Ho Hospital. These samples, originally collected for clinical diagnostics, were fully de−identified. The Institutional Review Board reviewed and addressed a related non−compliance (NC) event, confirming that appropriate corrective actions were taken in line with ethical and regulatory standards.”

The original version of this article has been updated.

