# Supplementary figures and images for: MED10 Drives the Oncogenicity and Refractory Phenotype of Bladder Urothelial Carcinoma Through the Upregulation of hsa-miR-590
Source: Front Oncol. 2022 Jan 13;11:744937. doi: 10.3389/fonc.2021.744937 (PMC8792749; doi:10.3389/fonc.2021.744937)

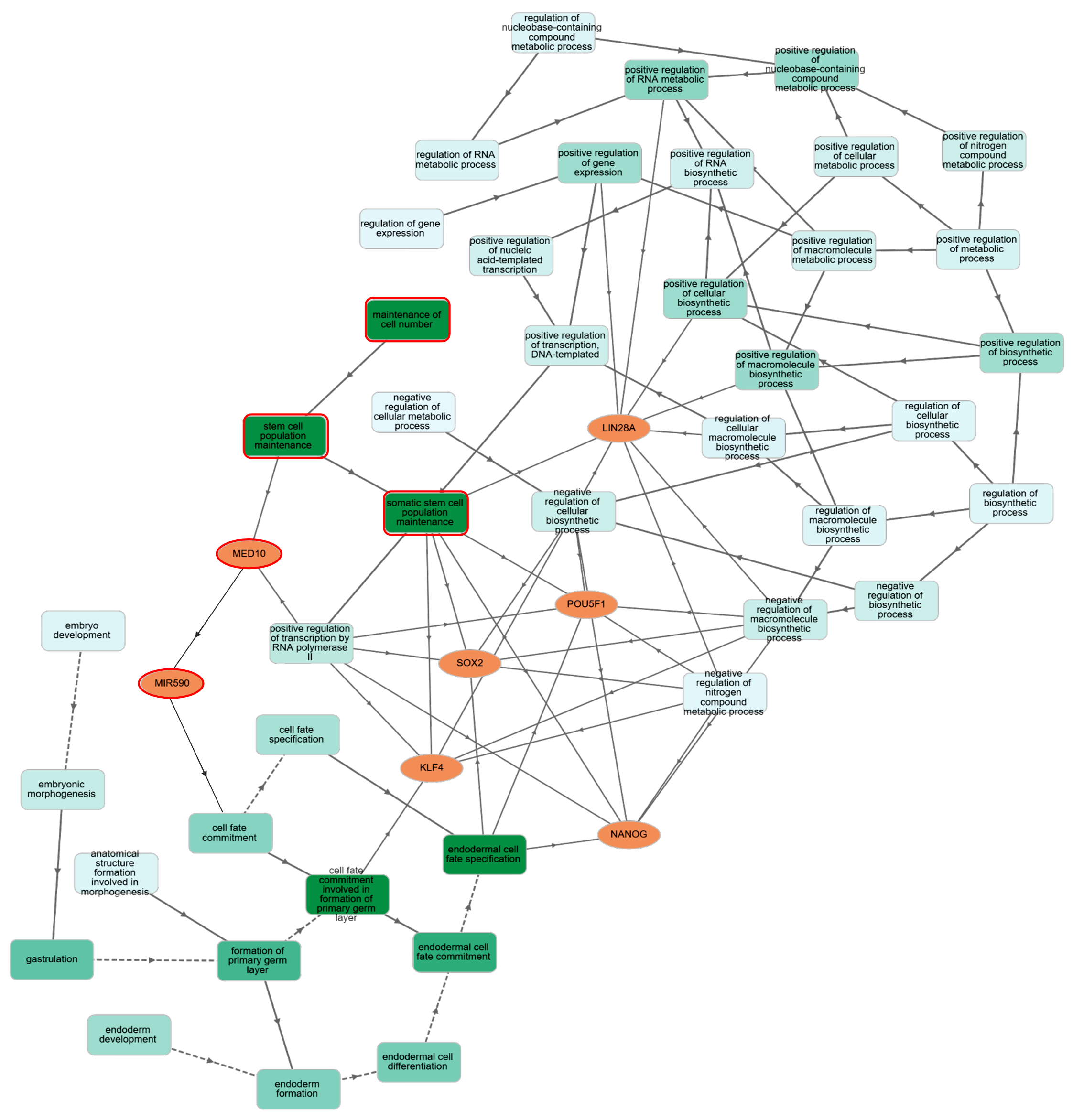

Supplement: Supplementary Figure 1 — The MED10/MIR590 signaling plays a critical role in stem cell population maintenance and cell fate determination. GO term enrichment was based on biological function. GO, gene ontology. [file Image_1.png]
